# Effect of soil microorganisms and labile C availability on soil respiration in response to litter inputs in forest ecosystems: A meta‐analysis

**DOI:** 10.1002/ece3.6965

**Published:** 2020-10-31

**Authors:** Yanjun Zhang, Junliang Zou, Delong Meng, Shuina Dang, Jinhong Zhou, Bruce Osborne, Yuanyuan Ren, Ting Liang, Keke Yu

**Affiliations:** ^1^ Geography and Environmental Engineering Department Baoji University of Arts and Sciences Baoji China; ^2^ Beijing Research & Development Centre for Grass and Environment Beijing Academy of Agriculture and Forestry Sciences Beijing China; ^3^ Key Laboratory of Biometallurgy Ministry of Education School of Minerals Processing and Bioengineering Central South University Changsha China; ^4^ School of Education Baoji University of Arts and Sciences Baoji China; ^5^ UCD School of Biology and Environmental Science and UCD Earth Institute University College Dublin Belfield, Dublin 4 Ireland

**Keywords:** labile C availability, litter inputs, meta‐analysis, soil microorganisms, soil respiration

## Abstract

Litter inputs can influence soil respiration directly through labile C availability and, indirectly, through the activity of soil microorganisms and modifications in soil microclimate; however, their relative contributions and the magnitude of any effect remain poorly understood. We synthesized 66 recently published papers on forest ecosystems using a meta‐analysis approach to investigate the effect of litter inputs on soil respiration and the underlying mechanisms involved. Our results showed that litter inputs had a strong positive impact on soil respiration, labile C availability, and the abundance of soil microorganisms, with less of an impact related to soil moisture and temperature. Overall, soil respiration was increased by 36% and 55%, respectively, in response to natural and doubled litter inputs. The increase in soil respiration induced by litter inputs showed a tendency for coniferous forests (50.7%)> broad‐leaved forests (41.3%)> mixed forests (31.9%). This stimulation effect also depended on stand age with 30‐ to 100‐year‐old forests (53.3%) and ≥100‐year‐old forests (50.2%) both 1.5 times larger than ≤30‐year‐old forests (34.5%). Soil microbial biomass carbon and soil dissolved organic carbon increased by 21.0%‐33.6% and 60.3%‐87.7%, respectively, in response to natural and doubled litter inputs, while soil respiration increased linearly with corresponding increases in soil microbial biomass carbon and soil dissolved organic carbon. Natural and doubled litter inputs increased the total phospholipid fatty acid (PLFA) content by 6.6% and 19.7%, respectively, but decreased the fungal/bacterial PLFA ratio by 26.9% and 18.7%, respectively. Soil respiration also increased linearly with increases in total PLFA and decreased linearly with decreases in the fungal/bacterial PLFA ratio. The contribution of litter inputs to an increase in soil respiration showed a trend of total PLFA > fungal/bacterial PLFA ratio > soil dissolved organic carbon > soil microbial biomass carbon. Therefore, in addition to forest type and stand age, labile C availability and soil microorganisms are also important factors that influence soil respiration in response to litter inputs, with soil microorganisms being more important than labile C availability.

## Graphical abstract

1. Soil respiration, labile C availability, and soil microorganisms (e.g., microbial quantity and community structure) were significantly influenced by litter inputs.
2. The increase in soil respiration in response to litter inputs was closely related to labile C availability and soil microorganisms.
3. Soil microorganisms had a greater effect than labile C availability influence soil respiration in response to litter inputs.

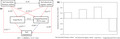

## 1 INTRODUCTION

Soils release approximately 98 Pg C to the atmosphere through soil respiration each year (Ben & Allison, 2010), which is ten times the rate of carbon emission by fossil fuel combustion (IPCC, 2013). Rates of soil respiration have been increasing by approximately 0.1 Pg C yr^−1^ since 1989 in response to global temperature increases (Ben & Allison, 2010), and small changes in soil respiration associated with climate change have the potential to influence atmospheric CO_2_ concentrations due to the large amounts of C stored in soils (Ben & Allison, 2010). Although extensive work has reported that soil respiration in forest ecosystems could be greatly affected by abiotic and biotic factors, such as soil temperature and moisture, soil microorganisms, and substrate supply (Fang et al., 2015; Tian et al., 2019; Wu et al., 2017), there is still uncertainty about how substrate supply, soil microbial community, soil temperature, and soil moisture interactively affect soil respiration under field conditions.

Although a number of studies have reported that litter inputs significantly affect soil respiration, this can vary with other factors, such as vegetation type (Duan et al., 2018; Han et al., 2015), successional stage (Han et al., 2015), stand age (Xin et al., 2016), experimental study period (Crow et al., 2009; Sayer, 2006; Wang et al., 2009, 2013), climatic conditions (Deng et al., 2007; Liang et al., 2010; Sulzman et al., 2005; Zhang et al., 2016; Zimmermann et al., 2009), the quantity and quality of litter (Bréchet et al., 2018; Deng et al., 2007; Duan et al., 2018), topography (Duan et al., 2018; Zhang et al., 2020), soil temperature and moisture (Fekete et al., 2014; Sulzman et al., 2005), and soil physicochemical properties (e.g., soil pH, soil C:N, soil bulk density) (Pinto et al., 2018; Zhang et al., 2020). In addition, the response of soil respiration to litter inputs can also be influenced by soil microorganisms (e.g., microbial quantity and community structure) (Han et al., 2015; Leitner et al., 2016; Wu et al., 2017). However, the effect of litter inputs on soil respiration and soil microorganisms is extremely complex. Most studies have found that soil respiration was significantly increased by litter inputs (Bréchet et al., 2018; Kim et al., 2005; Pinto et al., 2018; Sayer et al., 2007; Sulzman et al., 2005; Zhang et al., 2016, 2020; Zimmermann et al., 2009), while only a few have reported that soil respiration was not increased by litter inputs (Fekete et al., 2014; Sun et al., 2005). In contrast, soil microbial biomass has been found to increase (Wu et al., 2017), decrease (Leitner et al., 2016; Wang et al., 2013), or remain unchanged (Leitner et al., 2016) in response to litter inputs. Similarly, bacterial/fungal PLFA ratio may be decreased (Wu et al., 2017) or increased (Wang et al., 2013) in response to litter inputs.

The Detritus Input and Removal Treatment (DIRT) experiment provides a unique opportunity to examine feedbacks between litter inputs, soil microorganisms, and soil respiration through long‐term manipulation of aboveground litter inputs in forest ecosystems (Bréchet et al., 2018; Sulzman et al., 2005; Veres et al., 2015; Wu et al., 2017; Zhang, 2017). While there are some review articles about the effects of litter inputs on soil physicochemical and biological processes (Xu et al., 2013), such as soil respiration (Chen & Chen, 2018; Lv & Wang, 2017; Zhang et al., 2020) and there is information from field experiments on the relationships among litter inputs, soil microorganisms and soil respiration (Han et al., 2015; Leff et al., 2012; Leitner et al., 2016; Wang et al., 2013; Wu et al., 2017), the results are variable and the generality of the findings are unclear as they lack regional representation. Litter inputs can influence soil respiration directly through an increase in labile C availability and, indirectly, through the activity of soil microorganisms and modifications in soil moisture and temperature (Han et al., 2015; Leitner et al., 2016; Sulzman et al., 2005; Wu et al., 2017). However, our current understanding of the interrelationships among litter inputs, soil microorganisms, and soil respiration is extremely limited. More importantly, how labile C availability and soil microorganisms, directly or indirectly, drive soil respiration, as well as their relative contributions, remains poorly understood. Therefore, the primary objectives of this study were: (1) to examine how soil respiration, labile C availability, and soil microorganisms respond to altered litter inputs and (2) to quantify the relative contributions of labile C availability and soil microorganisms to soil respiration in response to litter inputs. To achieve these goals, we conducted a meta‐analysis of 66 recent studies where there was long‐term manipulation of aboveground litter inputs in forest ecosystems where changes in soil respiration were investigated.

## 2 METHODS

### 2.1 Data selection

Data were extracted from peer‐reviewed publications that reported on soil respiration in both treatment plots (receiving litter inputs) and control plots (no‐litter inputs). The relevant publications were selected via searching keywords using the terms “litter respiration”, “contribution of litter respiration to soil respiration”, “effect of litter on soil respiration”, and “temperature sensitivity of litter respiration”. These terms were used in searches of the Web of Science and the China Knowledge Resource Integrated Database (CNKI). Studies lacking replication in their experimental design (e.g., Berryman et al., 2013; Cisneros‐Dozal et al., 2007; Kim et al., 2005; Liang et al., 2010; Ngao et al., 2005) were excluded. Papers with no natural litter inputs (e.g., Fang et al., 2015) were also excluded. If an article only reported the standard error, the standard deviation was calculated through the following equation:

(1) $$SD=SE\sqrt{N}$$

where *N* = number of replicates.

To conduct a comprehensive analysis, the final dataset comprised 66 studies (Table S1) conducted between 1989 and 2020, including 2,436 observations of which 1543 observations were from broad‐leaved forest, 408 observations were from coniferous forest, and 485 observations were from mixed forest.

### 2.2 Meta‐analysis

The raw data were either obtained from tables or extracted by digitizing graphs using the GetData Graph Digitizer (version 2.24, Russian Federation). For each paper, the following information was compiled: source(s) of data, location (e.g., longitude, latitude, and altitude), climatic information (e.g., mean annual temperature and precipitation), vegetation type (e.g., coniferous forest, broad‐leaved forest, and mixed forest), stand age (e.g., ≤30‐year‐old forests, 30‐ to 100‐year‐old forests, and ≥ 100‐year‐old forests), soil microbial quantity (e.g., total PLFA), and community structure (e.g., fungal/bacterial PLFA ratio), soil microbial biomass carbon, soil dissolved organic carbon, soil temperature and moisture, and soil respiration.

The effect size for each investigation was calculated as the natural log‐transformed response ratio (lnRR):

(2) $$\ln RR=\frac{\ln X_{t}}{\ln X_{c}}=\ln X_{t}‐\ln X_{c}$$

where RR is the response ratio, *X_t_* is the mean soil respiration in the plots receiving litter, and *X_c_* is the mean soil respiration without litter. The weighted mean effect size (*RR*_++_) for each categorical subdivision was calculated, and a bias‐corrected 95% confidence interval (CI) was determined by applying a bootstrapping procedure using MetaWin 2.1 (Sinauer Associates) (Hedges et al., 1999; Luo et al., 2006). The detailed calculation of the weight (w) and variance (v) of each RR and the weighted mean effect size (*RR*_++_) were described as detailed in Zhou et al. (2014) and Zhou et al. (2016). The effect of litter inputs on soil respiration within a categorical subdivision was considered significant at *p* < .05 if the 95% CIs did not include 0. In addition, the increase in soil respiration (%) was calculated using the following formula (Chang et al., 2014):

(3) $$increase\left(\%\right)=\left(e^{RR_{++}}‐1\right)\times 100\%$$

Statistical analyses (relationships among the increase in soil respiration and the increase in labile C availability and soil microorganisms) were performed using SigmaPlot 10.0 software (Systat Software, Inc.). Additionally, the structural equation model was used to discriminate the direct and indirect influence of soil microorganisms (e.g., total PLFA and fungal/bacterial PLFA ratio) and labile C availability (e.g., soil microbial biomass carbon and soil dissolved organic carbon) on soil respiration in response to litter inputs using AMOS 20.0 (AMOS IBM, USA).

## 3 RESULTS

### 3.1 Effect of litter inputs on soil respiration

**Figure 1:**
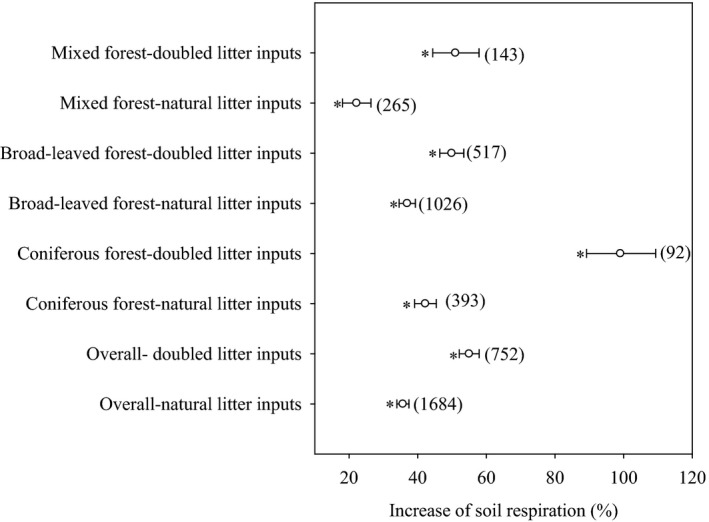
Effect of litter inputs on soil respiration. Numbers in brackets are the corresponding number of observations. Dots with error bars denote the overall mean percentage increase and its 95% CI. *denotes significant differences at *p* < .05

Soil respiration was increased significantly by litter inputs (Figure 1, *p* < .05), and the effect size was normally distributed (Figure S1). Overall, soil respiration increased by 35.7% and 55.0% in response to natural and doubled litter inputs, respectively (Figure 1). Respiration from the coniferous forest soil was increased by 42.2% and 99.1% in response to natural and doubled litter inputs, respectively, while respiration from the broad‐leaved forest soil was increased by 36.9% and 49.9% in response to natural and doubled litter inputs, respectively (Figure 1). For the mixed forest, soil respiration was increased by 22.2% and 51.0% in response to natural and doubled litter inputs, respectively (Figure 1). Our results clearly suggest that the increase in respiration induced by a doubling of the litter inputs is greater than the increase in respiration caused by natural litter inputs, which may exhibit a positive priming effect; the priming effect followed the order of coniferous forest > mixed forest > broad‐leaved forest.

### 3.2 Effect of labile C availability on soil respiration in response to litter inputs

**Figure 2:**
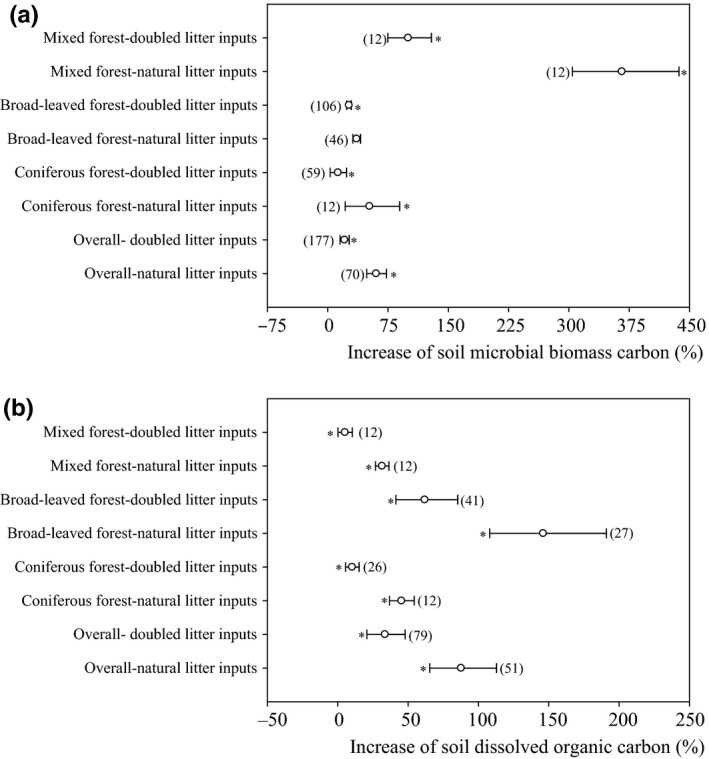
Effect of litter inputs on soil microbial biomass carbon (a) and soil dissolved organic carbon (b). Numbers in brackets are the corresponding number of observations. Dots with error bars denote the overall mean percentage increase and its 95% CI. *denotes significant differences at *p* < .05

**Figure 3:**
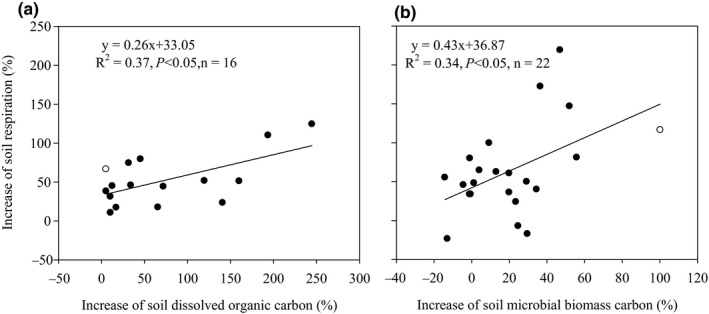
Relationships between the increases in soil dissolved organic carbon (a) and soil microbial biomass carbon (b) and the increase in soil respiration

Labile C availability (e.g., soil microbial biomass carbon and soil dissolved organic carbon) was increased significantly by litter inputs (Figure 2, *p* < .05). Overall, soil microbial biomass carbon was increased by 21.0% and 60.3% in response to natural and doubled litter inputs, respectively (Figure 2a). For the coniferous forest, broad‐leaved forest, and mixed forest, soil microbial biomass carbon was increased by 12.9% to 366.0% in response to natural and doubled litter inputs (Figure 2a). Overall, soil dissolved organic carbon was increased by 33.6% and 87.7% in response to natural and doubled litter inputs (Figure 2b). For the coniferous forest, broad‐leaved forest, and mixed forest, soil dissolved organic carbon was increased by 10.3% to 146.0% in response to natural and doubled litter inputs (Figure 2b). Soil respiration increased linearly with litter input and corresponding increases in soil dissolved organic carbon and soil microbial biomass carbon (Figure 3a, b).

### 3.3 Effect of soil microorganisms on soil respiration in response to litter inputs

**Figure 4:**
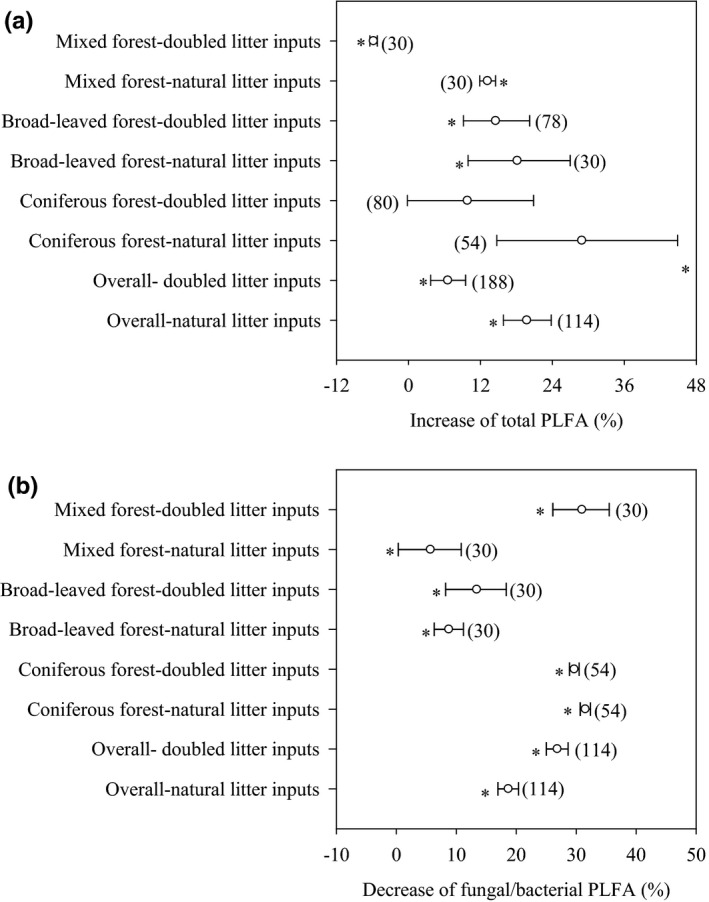
Effect of litter inputs on total PLFA (a) and fungal/bacterial PLFA ratio (b). Numbers in brackets are the corresponding number of observations. Dots with error bars denote the overall mean percentage increase and its 95% CI. *denotes significant differences at *p* < .05

**Figure 5:**
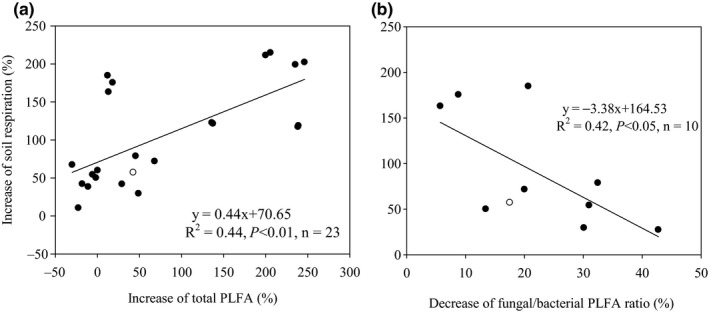
Relationships between the increases in total PLFA (a) and fungal/bacterial PLFA ratio (b) and the increase in soil respiration

**Figure 6:**
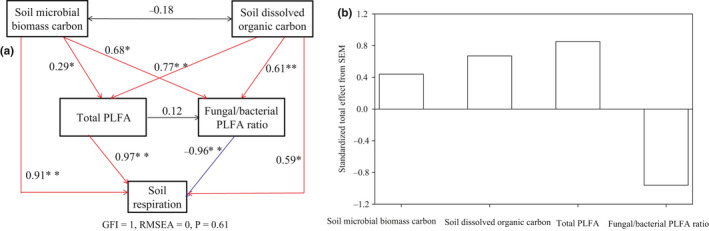
Structural equation model evaluating the direct and indirect effects of soil microbial biomass carbon, soil dissolved organic carbon, total PLFA, and fungal/bacterial PLFA ratio on soil respiration (a) and the standardized total effect (direct plus indirect effects) of these factors derived from the structural equation model (b) in forest ecosystems. Red and blue lines indicate positive and negative relationships, respectively; black lines indicate the relationship is not significant at *p* < .05 level; Numbers adjacent to arrows are standardized path coefficients, indicating the effect size of the relationship. * denotes significant differences at *p* < .05, ** denotes significant differences at *p* < .01

Soil microorganisms (e.g., microbial quantity and community structure) were influenced significantly by litter inputs (Figure 4, *p* < .05). Overall, the total PLFA increased by 6.6% and 19.7% in response to natural and doubled litter inputs, respectively (Figure 4a). Total PLFA from the coniferous forest and broad‐leaved forest were increased by 9.8% to 28.9% in response to natural and doubled litter inputs, while the total PLFA from the mixed forest was decreased by 5.83% in response to natural litter inputs and increased by 13.2% in response to doubled litter inputs (Figure 4a). Overall, the fungal/bacterial PLFA ratio was decreased by 26.9% and 18.7% in response to natural and doubled litter inputs, respectively (Figure 4d). The fungal/bacterial PLFA ratio for the coniferous forest, broad‐leaved forest, and mixed forest decreased by 5.7% to 31.5% in response to natural and doubled litter inputs (Figure 4d). Similar to the data for labile carbon, soil respiration increased linearly with corresponding increases in total PLFA and decreased linearly with corresponding decreases in fungal/bacterial PLFA ratio (Figure 5a, b). Furthermore, the structural equation model clearly showed that the variable effects of these factors (e.g., dissolved organic carbon, soil microbial biomass carbon, total PLFA, and fungal/bacterial PLFA ratio) on soil respiration in response to litter inputs (Figure 6a). Assessment of the contribution of litter inputs to the increase in soil respiration showed that the fungal/bacterial PLFA ratio had the greatest effect, followed by the total PLFA and soil dissolved organic carbon, with soil microbial biomass carbon having the smallest effect, as indicated by the standardized total effects obtained from the structural equation model (Figure 6b).

### 3.4 Effect of soil temperature and soil moisture on soil respiration in response to litter inputs

**Figure 7:**
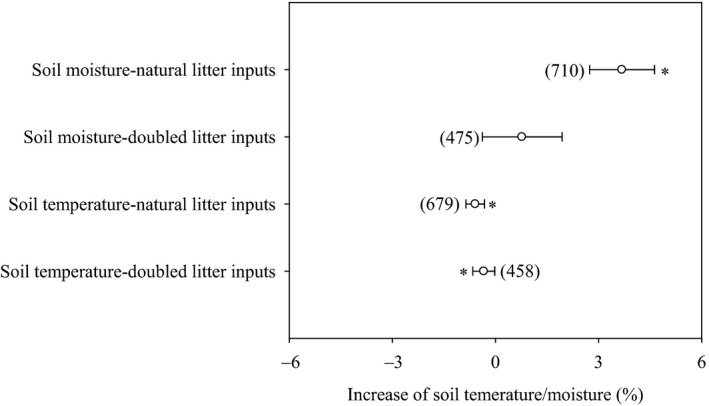
Effect of litter inputs on soil temperature and soil moisture. Numbers in brackets are the corresponding number of observations. Dots with error bars denote the overall mean percentage increase and its 95% CI. *denotes significant differences at *p* < .05

**Figure 8:**
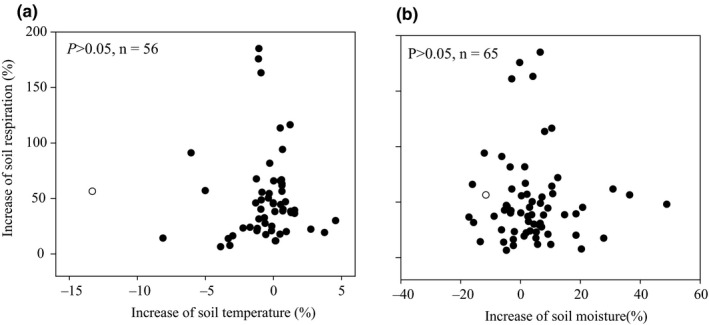
Relationships between the increases in soil temperature (a), soil moisture (b) and the increase in soil respiration

Soil temperature and soil moisture were influenced significantly by litter inputs (Figure 7, *p* < .05). Soil temperature was decreased by 0.6% and 0.3% in response to natural and doubled litter inputs, respectively (Figure 7). Soil moisture was not influenced by doubled litter inputs but increased by 3.7% with natural litter inputs (Figure 7). Increases in soil respiration in response to litter inputs were, however, unrelated to changes in soil temperature and soil moisture (Figure 8a, b; *p* > .05).

### 3.5 Effect of forest type and stand age on soil respiration in response to litter inputs

**Figure 9:**
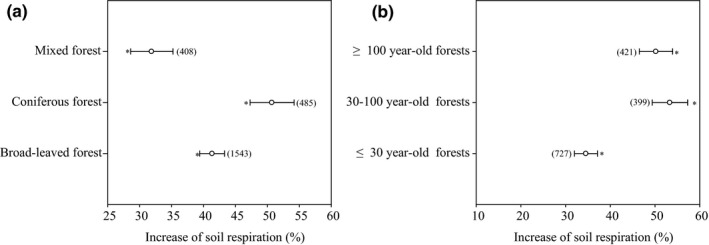
Effect of forest type and stand age on soil respiration in response to litter inputs. Numbers in brackets are the corresponding number of observations. Dots with error bars denote the overall mean percentage increase and its 95% CI. *denotes significant differences at *p* < .05

**Figure 10:**
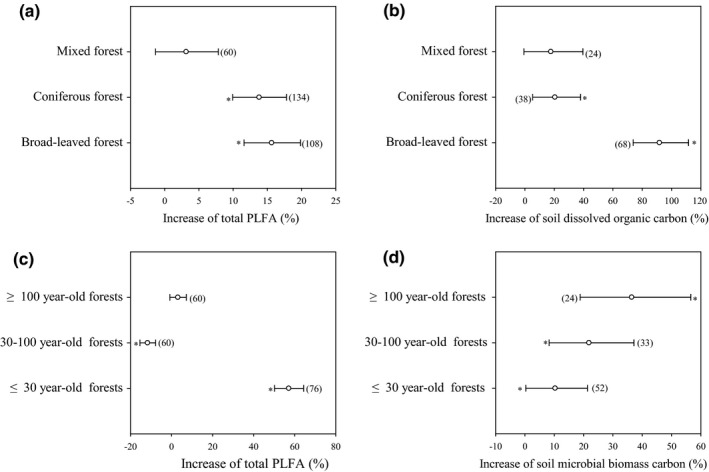
Effect of forest type on total PLFA (a) and soil dissolved organic carbon (b), and stand age on total PLFA (c), and soil microbial biomass carbon (d) in response to litter inputs. Numbers in brackets are the corresponding number of observations. Dots with error bars denote the overall mean percentage increase and its 95% CI *denotes significant differences at *p* < .05

The increase in soil respiration induced by litter inputs was significantly influenced by forest type and stand age (Figure 9a, b; *p* < .05). Soil respiration increased by 50.7% in coniferous forests, 41.3% in broad‐leaved forests, and 31.9% in mixed forests (Figure 9a). At the same time, the total PLFA increased by 13.8% and 15.6% in coniferous and broad‐leaved forests, whereas it only increased (statistically insignificant) by 3.1% in mixed forests (Figure 10). Corresponding values for soil dissolved organic carbon showed that these increased by 20.5% and 91.8% in coniferous and broad‐leaved forests, whereas it only increased by 17.7% in mixed forests (Figure 10b). Therefore, the effect of forest type on soil respiration seemed to be related to the increase in soil dissolved organic carbon and total PLFA associated with litter inputs.

In terms of stand age, the largest response of soil respiration to litter inputs occurred in 30‐ to 100‐year‐old forests (53.3%) and ≥100‐year‐old forests (50.2%) (Figure 9b). However, the total PLFA decreased by 11.7% in 30‐ to 100‐year‐old forests and only increased (statistically insignificant) by 3.1% in ≥100‐year‐old forests, whereas it increased by 57.1% in ≤ 30‐year‐old forests (Figure 10 c). For soil microbial biomass carbon, there was an increase of 21.9% and 31.4% in 30‐ to 100‐year‐old and ≥100‐year‐old forests, whereas it only increased by 10.3% in ≤30‐year‐old forests (Figure 10 d). Therefore, the effect of stand age on soil respiration seemed to be related mainly to the increase in soil microbial biomass carbon associated with litter inputs.

## 4 DISCUSSION

### 4.1 Effect of litter inputs on labile C availability and soil respiration

Our results showed that soil respiration was increased, on average, by 35.7% in response to natural litter inputs, while a doubling of litter inputs increased soil respiration by 55.0%, consistent with previous field studies (Bréchet et al., 2018; Sayer et al., 2007; Sulzman et al., 2005; Zhang et al., 2016). Our results suggesting that the increase in respiration induced by a doubling of litter inputs is greater than the increase in respiration caused by natural litter inputs, which may exhibit a positive priming effect, is also consistent with previous studies (Bréchet et al., 2018; Chen & Chen, 2018; Sayer et al., 2007; Sulzman et al., 2005; Zhang et al., 2014). Clearly, this could be due to the increase in labile C availability (e.g., increased soil dissolved organic carbon and soil microbial biomass carbon) associated with litter inputs. Substantial increases in the availability of labile C for soil microorganisms would result in a stimulation of soil respiration if this is limited by substrate availability (Klotzbücher et al., 2012; Kuzyakov & Blagodatskaya, 2015). Similar to other work (Leff et al., 2012; Leitner et al., 2016; Liu et al., 2017; Wang et al., 2013), our study showed that soil dissolved organic carbon and soil microbial biomass carbon were increased by 33.6%–87.7% and 21.0%–60.3% in response to litter inputs, respectively (Figure 3c). An increase in respiration due to litter‐related enhanced substrate availability is also supported by the positive correlation between soil respiration and an increase in soil dissolved organic carbon and soil microbial biomass carbon (Figure 4). Therefore, differences in labile C availability (e.g., soil dissolved organic carbon and soil microbial biomass carbon) due to varying litter inputs may explain some differences in soil respiration.

### 4.2 Effect of soil microorganisms on soil respiration in response to litter inputs

Litter inputs may also have elicited changes in soil respiration indirectly by affecting both the total numbers and population structure of soil microorganisms (Leff et al., 2012). Our results showed that the total PLFA was significantly increased, while the fungal/bacterial PLFA ratio was significantly decreased at both high and low litter inputs (Figure 5). This suggest that increased labile C availability, or other biological or physical factors associated with litter inputs, favored the growth of some microbial groups over others, resulting in shifts in the microbial community (Brant et al., 2006; Nadelhoffer et al., 2004; Strickland et al., 2009; Wang et al., 2013; Wu et al., 2017; Yan et al., 2018). However, increasing litter inputs can have different effects on the soil microbial community composition and quantity, depending on the forest type and season. For example, in a temperate beech forest in Austria the total PLFA increased by 29% in August, decreased by 12% in October, and remained largely unchanged in December in response to natural litter inputs (Leitner et al., 2016). Another study conducted in three successional subtropical forests in southern China showed that litter exclusion significantly increased the fungal PLFA and the fungal/bacterial PLFA ratio (Han et al., 2015). While litter additions significantly increased the total PLFA in a coniferous and a mixed forest, in a broadleaf forest the soil microbial community was not altered by either litter exclusion or litter addition (Han et al., 2015).

We also found that the increase in soil respiration was closely correlated with the increase in total PLFA (Figure 5a), suggesting that changes in soil microbial biomass in response to litter inputs may explain some variations in soil respiration, results that are similar to earlier studies (Feng et al., 2009; Han et al., 2015; Leitner et al., 2016; Li et al., 2004; Wang et al., 2013; Wu et al., 2017). In a coniferous forest ecosystem in central China, basal soil respiration was also positively correlated with total PLFA in response to litter inputs (Wu et al., 2017). Fungi and bacteria differ in their strategies for using C, with fungi characterized by a low respiration quotient and a higher efficiency in their use of C as they produce more biomass C per unit of C metabolized than do bacteria (Deng et al., 2016; Strickland & Rousk, 2010). In response to litter inputs, we found that the increase in soil respiration was closely correlated with a decrease in the fungal/bacterial PLFA ratio (Figure 5d), suggesting that alterations in the relative abundance of fungi and bacteria in response to litter inputs may explain some variations in soil respiration (Han et al., 2015; Wang et al., 2013; Wu et al., 2017). Similarly, in a coniferous forest ecosystem of central China, basal soil respiration was negatively correlated with fungal/bacterial PLFA ratio (Wu et al., 2017). Therefore, both labile C availability (e.g., soil dissolved organic carbon and soil microbial biomass carbon) and differences in the soil microbial community may also contribute to changes in soil respiration. The contribution of labile C availability and microbial community composition to the increase in soil respiration followed the order total PLFA > fungal/bacterial PLFA ratio > soil dissolved organic carbon > soil microbial biomass carbon (Figure 6), which suggests that soil microorganisms are more important than labile C availability in influencing litter‐related increases in soil respiration.

### 4.3 Effect of soil temperature and moisture on soil respiration in response to litter inputs

Litter inputs also indirectly influence soil temperature and soil moisture through their shading effects on soil temperature and the infiltration and evaporation of water (Fekete et al., 2014; Han et al., 2015; Sulzman et al., 2005; Zhang et al., 2014). Soil temperature was decreased by 0.3%‐0.6% in response to litter inputs, and soil moisture was increased by 3.7% (Figure 8). However, the effect of litter inputs on soil temperature and soil moisture can vary with vegetation type (Han et al., 2015; Zhao et al., 2014) and climate (Sayer & Tanner, 2010; Wu et al., 2017; Zhang, 2017). Though the soil temperature and soil moisture were influenced to some extent by litter inputs (Figure 8), no significant relationships were found between the increase in soil respiration and soil temperature or soil moisture (Figure 9, *p* > .05). Therefore, any changes in soil temperature and soil moisture due to litter inputs over the range examined are unlikely to have any significant impacts on soil respiration.

### 4.4 Effect of forest type and stand age on soil respiration in response to litter inputs

Similar to other studies (Deng et al., 2007; Yan et al., 2013; Han al., 2015), forest type had a significant impact on soil respiration in response to litter inputs. Litter‐associated increases in soil respiration in coniferous and broad‐leaved forests were 58.9% and 29.5% higher than that from mixed forests (Figure 9a), which could be attributed to the change in both total PLFA and labile C availability. This is likely due to differences in litter quality as different forest types produce variable amounts of litter with different chemical compositions (Deng et al., 2007; Yan et al., 2013). Generally, coniferous forest litter has a higher C/N ratio and lignin content than broad‐leaved forest and mixed forest (Han et al., 2015), resulting in differing contributions to soil respiration, and the abundance, composition, and activity of soil microbial communities (Han et al., 2015; Sulzman et al., 2005). In our studies, the total PLFA content increased by 13.8% and 15.6% in coniferous and broad‐leaved forests, respectively, whereas it only increased by 3.1% in mixed forests (Figure 10a). For soil dissolved organic carbon, this increased by 20.5% and 91.8% in coniferous and broad‐leaved forests, whereas it only increased by 17.7% in mixed forests (Figure 10b).

Previous studies have shown that soil respiration may increase (Han et al., 2015; Yan et al., 2006), decrease (Wang et al., 2016), or remain unchanged (Xiao et al., 2014; Yu et al., 2016; Zhao et al., 2016) with stand age in response to litter inputs. The effect of stand age on litter‐related soil respiration has been attributed to changes in soil temperature and soil moisture (Han et al., 2015; Yu et al., 2016), substrate availability (e.g., soil organic carbon and soil microbial biomass carbon) (Xiao et al., 2014; Yu et al., 2016), or litter quantity and quality (Han et al., 2015; Yan et al., 2006; Zhao et al., 2016). In our studies, the effects of litter inputs on soil respiration increased with stand age, which was greater (45%–55%) in 30‐ to 100‐year‐old and ≥100‐year‐old forests compared to that in ≤30‐year‐old forests and was related to increased labile carbon availability (Figure 10d). This is because in response to litter inputs, 30‐ to 100‐year‐old and ≥100‐year‐old forests produced more labile carbon than ≤30‐year‐old forests, with soil microbial biomass carbon in the 30‐ to 100‐year‐old and ≥100‐year‐old forests 2–3 times larger than that in ≤30‐year‐old forests (Figure 10d). Similarly, in different‐aged (e.g., 20, 30, and 46 years old) *Pinus massoniana* forests in the three gorges reservoir area, litter respiration contributed 31.0%‐45.9% for the three different‐aged forests, with the lower contribution in the 30‐year‐old stands, which can be attributed to the lower soil organic matter and nitrogen contents, compared to that in the other two stands (Xiao et al., 2014).

## 5 CONCLUSIONS

In this paper, the effects of soil microorganisms (based on PLFA analysis), soil temperature and soil moisture, labile C availability, forest type, and stand age on soil respiration, in response to litter inputs, were analyzed through a meta‐analysis. Soil respiration, labile C availability, and soil microorganisms (e.g., microbial quantity and community structure) were significantly influenced by litter inputs. In response to litter inputs, the increase in soil respiration was closely related to modifications in the soil microbial community and labile C availability, with soil microorganisms having a greater effect than labile C availability. Similarly, changes in the soil microbial community and labile C availability were also associated with differences in soil respiration due to forest type or stand age. This suggests that the major driver of litter‐related increases in soil respiration is the associated changes in soil microbial populations. This will, in turn, depend mainly on litter quality and associated decomposition processes that release labile carbon to the soil, while any effects of litter inputs through modifications in soil microclimate would be expected to be small.

## CONFLICT OF INTEREST

I would like to declare on behalf of my co‐authors that no conflict of interest exits in the submission of this manuscript, and the manuscript is approved by all authors for publication.

## AUTHOR CONTRIBUTION

**Yanjun Zhang:** Writing‐original draft (equal). **Junliang Zou:** Methodology (equal). **Delong Meng:** Funding acquisition (equal). **Shuina Dang:** Data curation (equal). **Jinhong Zhou:** Methodology (equal). **Bruce Osborne:** Writing‐review & editing (equal). **Yuanyuan Ren:** Data curation (equal). **Ting Liang:** Conceptualization (equal). **Keke Yu:** Validation (equal).

## Supporting information

Figure S1

Table S1

## Data Availability

No data are associated with this manuscript.
