# Supplementary material for: Effect of soil microorganisms and labile C availability on soil respiration in response to litter inputs in forest ecosystems: A meta‐analysis
Source: Ecol Evol. 2020 Oct 31;10(24):13602–12. doi: 10.1002/ece3.6965 (PMC7771185; doi:10.1002/ece3.6965)
Supplement: Supplementary file 1 — Figure S1 [file ECE3-10-13602-s001.docx]

**Figure legends**

Fig. S1 Normal distribution of the effect of litter inputs on soil respiration (effect size).
