# Supplementary material for: Effect of soil microorganisms and labile C availability on soil respiration in response to litter inputs in forest ecosystems: A meta‐analysis
Source: Ecol Evol. 2020 Oct 31;10(24):13602–12. doi: 10.1002/ece3.6965 (PMC7771185; doi:10.1002/ece3.6965)
Supplement: Supplementary file 2 — Table S1 [file ECE3-10-13602-s002.docx]

**Table S1** **List of references associated with the data analysis**

| References | Geographic location | MAP | MAT | Altitude/m | VZ |
| --- | --- | --- | --- | --- | --- |
| Zhang Yanjun, 2017 | 35°13'N, 107°40'E | 586 | 9.4 | 1095 | B |
| Ge et al., 2018 | 120°0'E, 30°06'N | 1513 | 16.9 | 141 | B |
| Bai et al., 2017 | 112°32'E, 36°44'N | 600 | 6.2 | 2281 | C |
| Chen et al., 2017 | 119.08°E, 26.21°N | 1674 | 17.15 | - | C |
| Xiong et al., 2015 | 102°56'E, 31°18'N | 850 | 3.0 | 3035 | C |
| Gao et al., 2017 | 117°24'E, 23°38'N | 1103.8 | 20.8 | - | M |
| Xie et al., 2017 | 109.32°E,36.85°N | 500 | 8.8 | 1198 | C,M |
| Tian et al., 2012 | 103°38'E, 29°95'N | 1510.7 | 16.0 | - | B |
| Peng et al., 2018 | 117°30' E, 36°46'N | 600 | 8.7 | 2315 | C |
| Duan et al., 2018 | 122°17'E, 53°24'N | 425 | -4.9 | 415 | C,M |
| Liu et al., 2008 | 115°36'E, 39°53'N | 563.4 | 10.2 | 706 | C,B |
| Chen et al., 2018 | 113°01'E, 28°01'N | 1422 | 17.2 | 80 | B |
| Xie et al., 2017 | 99°56'E, 38°26'N | 450 | 0.7 | 3083 | C |
| Wang et al., 2013 | 109°28'E, 19°32'N | 1750 | 23.6 | 134 | B |
| Lu et al., 2010 | 128°37'E, 48°07'N | 640.5 | -0.5 | 480 | C,B |
| Wang et al., 2016 | 115.5°25'E, 33°28'N | 900 | 15.1 | 1215 | B |
| Deng et al., 2018 | 112°10'E, 23°10'N | 1956 | 21.4 | 250 | B |
| Hu et al., 2017 | 114°04'E, 31°49'N | 1061.7 | 15.3 | 210 | C,B |
| Zimmermann  et al., 2009 | 71°35'E, 13°11'S | - | 12.5 | 3030 | B |
| Gora et al., 2018 | 79°50'E, 9°06'S | 2600 | 27 | 43 | B |
| Sulzman et al., 2005 | 122°10'W, 44°15'N | 1986 | 9.06 | 531 | C |
| Bréchet et al., 2018 | 9°06'W, 79°54'N  1°19'W, 51°46'N | 2600/714 | 27/10.0 | - | M |
| Deng et al., 2007 | 112°33'E, 23°10'N | 1956 | 20.8 | 200 | B,C,M |
| Osvaldo et al., 2018 | 56°24'W, 16°29'S | 1420 | 26.5 | 125 | M |
| Sayer et al., 2007 | 79°54'W, 9°06'N | 2600 | 27 | - | B |
| Liu et al., 2017 | 117°28'E, 26°09'N | 1906 | 18.7 | 300 | B |
| Wu et al., 2017 | 111°13'E, 32°45'N | 749.3 | 15.7 | 340 | C |
| Atarashi-Andoh  et al., 2012 | 140.56°E, 40.0°N | 1207 | 6.1 | 825 | B |
| Liu et al., 2014 | 114°15' E, 37°52' N | 560 | 13 | 514 | B |
| Duan et al., 2020 | 122°17'E, 53°24'N | 425 | -4.9 | 475 | C,M |
| Yan et al., 2013 | 113°03'E, 28°07'N | 1450 | 17.2 | 80 | C,M,B |
| Rey et al., 2002 | 11°55'E, 42°24'N | 755 | 14 | 140 | B |
| Xiao et al., 2014 | 110.5°09'E, 30.5°25'N | 1125 | 18 | 763 | C |
| Wang et al., 2009 | 115.06 E, 26.74° N, | 1485 | 17.9 | 92 | C |
| Bowden et al., 1992 | - | - | - | 325 | M |
| Li et al., 2004 | - | 3500 | 22.3 | - | B,C |
| Yan et al., 2006 | 112°32.5'E, 23°11.5'N | 1956 | 20.9 | 250 | C,M,B |
| Sayer et al., 2011 | 79°54′W, 09°06′S | 2600 | 27 | - | B |
| Luo et al., 2001 | 108°49'E, 18°40'N | 2651.6 | 19.7 | 950 | B |
| Gao et al., 2015 | 121°39'E, 29°52'N | 1374.7 | 16.2 | 200 | B |
| Wang et al., 2009 | 133°31'E, 47°35'N | 600 | 1.9 | 56 | B |
| Leff et al., 2012 | 83°37′W, 08°43′N | 4430 | 25 | - | B |
| Boone al., 1998 | 72°12′W, 42°30′N | 1100 | 6 | - | M |
| Wang et al., 2012 | 111.5°34'E，36.5°11.5'N | 662 | 8.6 | 1450 | C |
| Wang et al., 2009 | 113°34'E, 36.5°11.5'N | 1422 | 17.2 | 80 | B |
| Crow et al., 2009 | 112°13′W, 44°13′N | 2370 | 8.7 | 531 | C |
| Li et al., 2014 | 112°08'E, 36°37'N | 662 | 6.2 | 1619 | C |
| Lei et al., 2016 | 110°47'E, 30°59'N | 1125 | 16.9 | 1200-1240 | C |
| Li et al., 2016 | 117.60°E, 26.32°N | 1749 | 19.1 | 330 | B,C |
| Yu et al., 2014 | 117°59′E, 26°48′N | 1653 | 20 | 238 | B,C |
| Zhao et al., 2014 | 116°38′E, 41°38′N | 430.3 | 7.6 | 673.5-790.3 | B,C |
| Li et al., 2016 | 117°28′E, 26°11′N | 1670 | 20.1 | 310 | B |
| Sayer et al., 2010 | 79°54′W, 09°06′S | 2600 | 27 | - | B |
| Liu et al., 2013 | 112°24′E, 36°37.5′N | 662 | 8.6 | 1197-1208 | C |
| Chemidlin Prévost-Bouré et al., 2010 | 02°47′E, 48°29′N | 680 | 10.7 | 90 | B |
| Vasconcelos et al., 2009 | 47°57′W, 01°19′S | 2399-3179 | - | - | B |
| Leitner et al., 2016 | 16°17′E, 47°42′N | 796 | 6.5 | 600 | B |
| Fekete et al., 2014 | 20°26′E, 47°55′N | 599 | 10.8 | 325 | B |
| Yu et al., 2016 | 118°29.5′E, 41°11′N | 550 | 7.3 | 1010 | C |
| Berryman et al., 2014 | 116°50′W, 48°21′N | - | 3.7-6.6 | 854-1521 | M |
| Wang et al., 2013 | 109°17′E, 26°25′N | 1200 | 16.5 | 537.5 | C |
| Han et al., 2015 | 112°31.5′E, 23°10′N | 1680 | 22.3 | - | C,M,B |
| DeForest et al., 2009 | 83°50′W, 41°33′N | 840 | 9.2 | - | B |
| Zhao et al., 2016 | 116°43.5′E, 41°09′N | 430.3 | 7.6 | 660 | B |
| Wang et al., 2013 | 109°36′E, 26°50′N | 1200 | 16.5 | 650 | C |
| Wu et al., 2012 | 101°01′E, 24°32′N | 1947 | 11 | - | B |

MAP: Annual mean precipitation (mm), MAT: Annual mean temperature (℃), VZ: Vegetation zone, B: Broad-leaved forest, C: Coniferous forest, M: Mixed forest, G: Grass
